# γ-Oryzanols of North American Wild Rice (*Zizania palustris*)

**DOI:** 10.1007/s11746-013-2252-x

**Published:** 2013-04-25

**Authors:** Felix Aladedunye, Roman Przybylski, Magdalena Rudzinska, Dorota Klensporf-Pawlik

**Affiliations:** 1 Department of Chemistry and Biochemistry, University of Lethbridge, Lethbridge, Canada; 2 Max Rubner-Institut, Federal Research Institute for Nutrition and Food, Detmold, Germany; 3 Faculty of Food Science and Nutrition, Poznan University of Life Sciences, Poznan, Poland; 4 Department of Food Commodity Science, Poznan University of Economics, Poznan, Poland

**Keywords:** Wild rice, Regular brown rice, γ-Oryzanol, Hydroxylated steryl ferulate, Steryl caffeate, Steryl cinnamate

## Abstract

γ-Oryzanol, a natural mixture of ferulic acid esters of triterpene alcohols and sterols, are an important bioactive components present in rice bran oil. In light of the recent increase in the popularity of wild rice among consumers, and the possibility of a direct relationship between γ-oryzanol composition and its bioactivity, the oryzanol profile of major wild rice (*Zizania palustris*) grown in North America was studied and compared to regular brown rice (*Oryza sativa* L.). A total of twenty-three γ-oryzanol components were separated, identified and quantified by HPLC coupled to an Orbitrap MS. The distribution of individual γ-oryzanols was similar for all the wild rice but significantly different from those of the regular brown rice. Unlike in the regular brown rice, a significant amount of steryl caffeate and cinnamate were found in the wild rice samples. Generally, the amounts of γ-oryzanol in the wild rice were higher compared to the regular brown rice, 1,352 vs. 688 μg/g. The results from this study showed that wild rice had a more diverse γ-oryzanol composition and the higher amounts compared to the regular brown rice.

## Introduction

Rice (*Oryza sativa*) is the staple food for two-thirds of the world’s population [1]. In response to the expected world population growth, the International Rice Research Institute predicted that 800 million tons of rice will be required in 2025 [2]. With worldwide rice production currently less than the population growth rate, a significant increase in the consumption of wild rice (*Zizania* spp*.)* is expected. Indeed, the utilization of wild rice is gaining popularity among consumers, and it is grown and commonly available in the North American supermarkets and restaurants.

Rice contains many bioactive nutrients including: γ-oryzanol, phytic acid, tocopherols, tocotrienols, thiamine, riboflavin, niacin and folic acid. γ-Oryzanol, a mixture of ferulic acid esters of triterpene alcohols and phytosterols is chiefly responsible for many of the observed health benefits of rice and rice products [3]. γ-Oryzanol has been shown to possess antioxidant, anti-inflammatory, anti-tumor, and hypocholesterolemic activities [4–8], and has been approved for the treatment of nerve imbalances and menopausal disorders [9].

The origin, environmental factors, and genotype affects the composition, as well as the amount of individual components of γ-oryzanol in standard rice [10, 11]. Also, inconsistent data on the numbers and composition of individual components of γ-oryzanol are often reported in the literature depending on the analytical procedures employed [12]. Whereas the majority of the studies have focussed on the total amount of γ-oryzanol in rice products [13–17], a detailed profiling of the individual components is of paramount importance especially in the light of emerging evidence suggesting significant differences in the physiological activity of individual phytosterol ferulates. For instance, 24-methylenecycloartanyl ferulate has been shown to be a more efficient inhibitor of 2,2′-azobis(2-methylpropionamidine) dihydrochloride accelerated cholesterol oxidation in vitro than either cycloartenyl or campesteryl ferulates [18]. Also, dimethyl sterols, cycloartenyl and 24-methylenecycloartanyl were more effective than various 4,4′-desmethyl phytosterols against 12-*O*-tetradecanoylphorbol-13-acetate induced inflammatory activity [4].

Although several studies have been undertaken on the separation and quantification of γ-oryzanol in regular rice (*Oryza sativa*), to the best of our knowledge, no data are available on the oryzanol profile of North American wild rice (*Zizania palustris*). In a recently published study, Przybylski et al. [17] reported composition of lipid components of North America wild rice revealing that the lipids are an excellent source of essential fatty acids and significant amounts of nutraceuticals including γ-oryzanol, however, the γ-oryzanol content was quantified as a group of steryl ferulates. Thus, the main objective of the present study was to assess the composition and contribution of individual components of γ-oryzanol in North American commercial wild rice in comparison with regular brown rice.

## Materials and Methods

### Materials

Samples of commercial wild rice were obtained from the following suppliers, the abbreviation in the parentheses following the name of the rice is used henceforth in this paper: Minnesota Natural Lake (MNL; C & G Enterprises, MN, USA), Minnesota Naturally Grown Lake & River (MNGLR; Moose Lake Wild Rice Company, MN, USA), Minnesota Cultivated Wild Rice (MC; Moose Lake Wild Rice Company, MN, USA), Athabasca Alberta (AA; Alice Ptolemy Lakeland Wild Rice, Athabasca, AB, Canada), North Western Ontario (NOW; Shoal Lake Wild Rice, Winnipeg, MB, Canada), Manitoba Far North (FNM; Far North Wild Rice, MB, Canada), and Saskatchewan (S; Points North Wild Rice Company, Yorkton, SK, Canada). Regular medium (MGR) and long grain (LGR) brown rice were obtained from Riceland Foods (AR, USA) and used as references.

### Chemicals

Acetonitrile and isopropanol used in the study were of LC–MS grade and were obtained from Fisher Scientific Co. (Toronto, ON, Canada). Ultrapure deionized water was purified by a Nanopure Diamond laboratory water system (Barnstead, Dubuque, IA, USA). All other solvents and chemicals were of analytical grade and were purchased from Sigma-Aldrich (St. Louis, MO, USA). γ-Oryzanol was a kind gift from the Oryza Oil and Fat Chemical Co. Ltd. (Ichinomiya-City, Japan).

### Extraction of γ-Oryzanol

γ-Oryzanols were isolated from the rice samples utilizing sonic assisted methanol extraction (SAME) [19]. Briefly, ground wild rice kernels (1 g) were weighed into a threaded tube then 10 mL of methanol added and vortexed for 1 min, followed by sonication at 50 °C for 1 h. Extraction was repeated three times with fresh methanol and the combined extract was centrifuged at 5,000 rpm for 30 min. The supernatant was evaporated to 5 mL and subsequently analyzed by HPLC–MS.

### HPLC–MS

High performance liquid chromatography was carried out using an Accela HPLC system equipped with an Accela 1,250 pump and autosampler (Thermo Fischer Scientific, West Palm Beach, FL). The sample was separated at 25 °C on a Kinetex C18 column (2.6 μm; 150 × 3 mm; Phenomenex, MA) using a mobile phase consisting of acetonitrile, water and isopropanol with the following gradient:

**Table:** 

Time (min)	Acetonitrile	Water	Isopropanol	Flow rate (mL/min)
0	20	80	0	0.30
15	80	5	15	0.30
30	82	0	18	0.30
50	100	0	0	0.60
56	100	0	0	0.60
57	20	80	0	0.30
60	20	80	0	0.30

Injection volume was 10 μL and the UV detector was at 325 nm (Accela PDA). γ-Oryzanol components were identified with Exactive Orbitrap MS (Thermo Fischer Scientific, West Palm Beach, FL, USA).

The mass spectrometer was equipped with an ESI ion source, operated in both positive and negative mode. Xcalibur software was used for data acquisition and analysis. The mass spectrometer conditions were optimized for γ-oryzanol by infusion of an oryzanol standard at 5 μL/min into a 300 μL/min flow of mobile phase containing 85 % acetonitrile, 10 % isopropanol and 5 % water. The ESI ion source capillary temperature was set at 275 °C; the sheath gas flow rate at 25, and the auxiliary gas flow at 5. The capillary and tube lens voltages were at 55 and 110 V, respectively for positive mode, and −45 and −95 V, respectively, for negative mode. The spray voltage was +4.00 kV for positive mode and −3.97 kV for negative mode. Fragmentation of components was achieved by Higher Energy Collision Induced Dissociation (HCD) fragmentation at 100 eV. The spectra were collected in a range of 100–800 *m*/*z* at a scan rate of 1 scan/s. To prevent rapid contamination of the ion source by the crude extract, the first and the last 10 min of effluent from the HPLC were diverted to waste using a Rheodyne automated switching valve. Quantification of γ-oryzanol components was achieved by external calibration using the average areas of the six major steryl ferulates in the standard mixture (~98 % of total oryzanol), and assuming that the extinction coefficients for ferulic acid for all peaks were the same, following Britz et al. method [20].

### Statistical Analysis

Extraction of γ-oryzanols and HPLC–MS analyses were performed in triplicate and data are presented as means ± SD. Data were analyzed by single factor analysis of variance (ANOVA) and regression analysis using Minitab 2,000 statistical software (Minitab Inc. PA, ver. 15). Statistically significant differences between means were determined by Duncan’s multiple range tests for *P* ≤ 0.05.

### Results and Discussion

**Fig. 1:**
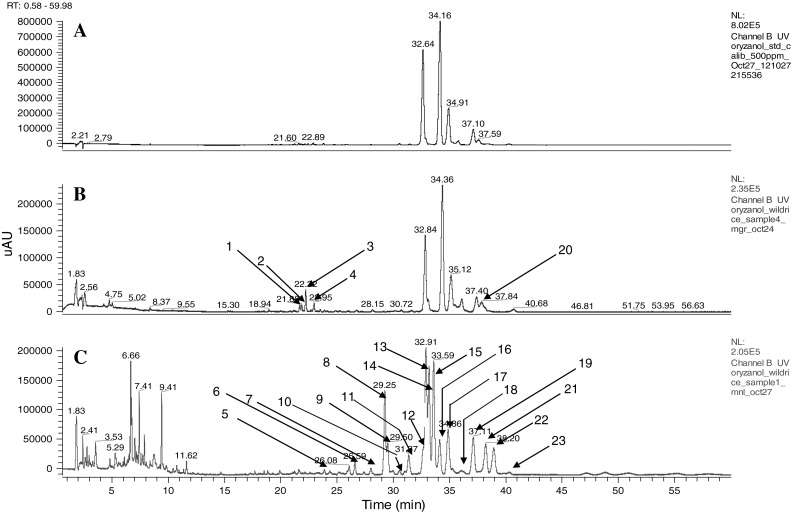
HPLC chromatograms of γ-oryzanol standards (**a**), regular Medium Grain and Long Grain brown rice (**b**), and Minnesota Natural Lake, MNL (**c**); similar patterns were observed for other samples of the wild rice. See Table 1 for identity of compounds

The chromatogram in Fig. 1 shows the twenty-three γ-oryzanol components separated in the present study, their identities are reported in Table 1. The identification of the components was based on the molecular mass determined by mass spectrometer, which agreed with the calculated values, and were also compared to the retention data of standards (chromatogram A; Fig. 1). In agreement with previously published data [18, 20, 21], cycloartenol *trans*-ferulate (*m*/*z* 601), 24-methylenecycloartanol *trans*-ferulate (*m*/*z* 615), campesterol *trans*-ferulate (*m*/*z* 575), and sitosterol *trans*-ferulate (*m*/*z* 589) were the major components, representing up to 90 % in the control brown rice and averaged of 75 % in the wild rice samples. Indeed, the amounts of 24-methylenecycloartanol *trans*-ferulate (249 μg/g), cycloartenol *trans*-ferulate (171 μg/g), and total γ-oryzanol (688 μg/g) obtained in the present study for long grain regular brown rice are in agreement with those reported by Cho et al. [21]. Significant differences, however, were observed in the contents of campesterol *trans*-ferulate and sitosterol *trans*-ferulate between the two studies, which could be due to differences in analytical procedures as earlier mentioned [12]. Fang et al. [12] identified three isomers of cycloartenol *trans*-ferulate in rice bran oil, in the present study; two isomers were identified in the control brown rice while four were identified in the wild rice samples (Fig. 1b, c).

**Table 1:** Composition and content of γ-oryzanol in wild and regular brown rice

Component (number on chromatogram)	[M–H]^−^	Amount (μg/g)								
		MNL	MNGLR	MC	AA	NOW	FNM	S	MGR	LGR
Hydroxy-24-methylenecycloartenol ferulate (1)	633.4150	0 ± 0^A^	0 ± 0^A^	0 ± 0^A^	0 ± 0^A^	0 ± 0^A^	0 ± 0^A^	0 ± 0^A^	1 ± 0	1 ± 0
Isomer of compound **1** (2)	633.4144	0 ± 0^A^	0 ± 0^A^	0 ± 0^A^	0 ± 0^A^	0 ± 0^A^	0 ± 0^A^	0 ± 0^A^	1 ± 0	1 ± 0
Hydroxycycloartenol ferulate (3)	617.4198	0 ± 0^A^	0 ± 0^A^	0 ± 0^A^	0 ± 0^A^	0 ± 0^A^	0 ± 0^A^	0 ± 0^A^	4 ± 1	3 ± 1
Isomer of compound **3** (4)	617.4198	1 ± 0	1 ± 0	1 ± 0	1 ± 0	1 ± 0	1 ± 0	1 ± 0	1 ± 0	1 ± 0
Hydroxydehydrocycloartenol ferulate (5)	615.4043	1 ± 0	1 ± 0	1 ± 0	1 ± 0	1 ± 0	1 ± 0	1 ± 0	0 ± 0^A^	0 ± 0^A^
Isomer of compound **1** (6)	633.4163	3 ± 1	3 ± 1	3 ± 1	3 ± 2	3 ± 1	2 ± 0	4 ± 1	0 ± 0^A^	0 ± 0^A^
Isomer of compound **3**	617.4187	1 ± 0	2 ± 1	1 ± 0	2 ± 1	2 ± 1	1 ± 0	2 ± 0	0 ± 0^A^	0 ± 0^A^
Cycloartenol caffeate? (8)	587.4100^B^	135 ± 9^a^	63 + 4^c^	95 + 7^b^	65 + 6^c^	68 + 4^c^	130 + 12^a^	68 + 5^c^	ND	ND
Campesterol caffeate? (9)	561.3943^B^	47 ± 3^a^	17 ± 2^c^	30 ± 2^b^	14 ± 2^c^	29 ± 3^b^	43 ± 2^a^	27 ± 2^b^	ND	ND
24-Methylenecholesterol ferulate (10)	573.3947	1 ± 0	1 ± 0	1 ± 0	1 ± 0	1 ± 0	1 ± 0	1 ± 0	1 ± 0	1 ± 0
Stigmasterol ferulate (11)	587.4106	47 ± 4^a^	31 ± 4^b^	40 ± 3^a^	34 ± 2^b^	31 ± 3^b^	45 ± 3^a^	32 ± 3^b^	8 ± 2^c^	6 ± 1^c^
Cycloartenol or cycloeucalenol ferulate (12)	601.4262	9 ± 1^a^	8 ± 1^a^	8 ± 2^a^	10 ± 2^a^	14 ± 2^b^	10 ± 1^a^	14 ± 2^b^	ND	ND
Cycloartenol ferulate (13)	601.4262	290 ± 22^a^	179 ± 11^b^	248 ± 20^ac^	232 ± 19^c^	269 ± 21^a^	286 ± 24^a^	286 ± 19^a^	196 ± 15^b^	171 ± 14^b^
Isomer of cycloartenol ferulate (14)	601.4263	202 ± 12^a^	68 ± 5^b^	56 ± 4^c^	53 ± 5^c^	57 ± 3^c^	192 ± 14^a^	56 ± 3^c^	32 ± 3^d^	28 ± 3^d^
Isomer of cycloartenol ferulate (15)	601.4262	142 ± 10^a^	100 ± 7^b^	17 ± 3^c^	77 ± 5^d^	78 ± 6^d^	154 ± 12^a^	77 ± 6^d^	ND	ND
24-Methylenecycloartenol ferulate (16)	615.4415	96 ± 11^a^	82 ± 6^abc^	75 ± 6^c^	79 ± 7^abc^	91 ± 7^ab^	94 ± 8^a^	92 ± 7^a^	367 ± 29^d^	249 ± 18^e^
Campesterol ferulate (17)	575.4104	123 ± 9^ab^	88 ± 6^c^	105 ± 8^d^	89 ± 8 ^cd^	95 ± 7^d^	125 ± 10^ab^	95 ± 8^d^	130 ± 9^a^	114 ± 8^b^
∆^7^-Sitosterol ferulate (18)	589.4258	2 ± 1^a^	1 ± 0^a^	1 ± 0^a^	2 ± 1^a^	1 ± 0^a^	1 ± 0^a^	1 ± 0^a^	47 ± 3^b^	38 ± 3^c^
Sitosterol ferulate (19)	589.4259	111 ± 9^a^	140 ± 11^b^	112 ± 9^a^	112 ± 7^a^	131 ± 8^b^	107 ± 10^a^	139 ± 8^b^	54 ± 4^c^	43 ± 3^d^
Cycloartanol ferulate (20)	603.4412	ND	ND	ND	ND	ND	ND	ND	54 ± 3^a^	14 ± 2^b^
Cycloartenol cinnamate? (21)	555.4410	71 ± 5^a^	37 ± 2^b^	50 ± 3^c^	37 ± 4^b^	45 ± 4^c^	77 ± 8^a^	47 ± 3^c^	ND	ND
Campesterol cinnamate? (22)	529.4258	70 ± 3^a^	45 ± 4^b^	75 ± 8^a^	36 ± 4^c^	37 ± 2^c^	60 ± 3^d^	36 ± 3^c^	ND	ND
Stigmastanol ferulate (23)	591.4418	1 ± 0^a^	2 ± 1^a^	1 ± 1^a^	2 ± 1^a^	2 ± 1^a^	1 ± 0^a^	2 ± 1^a^	20 ± 2^b^	18 ± 2^b^
Total		1352 ± 100^a^	867 ± 62^b^	920 ± 75^b^	850 ± 76^b^	956 ± 72^b^	1331 ± 107^a^	981 ± 71^b^	916 ± 69^b^	688 ± 55^c^

*MNL* Minnesota Natural Lake, *MNGLR* Minnesota Natural Grown Lake & River, *MC* Minnesota Cultivated Wild Rice, *AA* Athabasca Alberta, *NOW* North Western Ontario, *FNM* Manitoba far North, *S* Saskatchewan, *MGR* Medium Grain Rice, *LGR* Long Grain Rice, *ND* not detected

^A^Too small to be reliably quantified. Values with the same superscript within the same row are not significantly different

^B^not observed

The amount of total γ-oryzanol found in the wild rice samples ranged from 850 to 1,352 μg/g with MNL containing the highest and NOW the lowest amounts, these data are in agreement with a previous study by Przybylski et al. [17]. The higher amounts of γ-oryzanol reported in the present study compared to the previous study [17] may be due to the differences in extraction methods and the inclusion of better separation of new components: **8, 9, 21, 22** (Table 1) in the total amount of γ-oryzanol. Although some significant differences (*P* < 0.05) were observed among the wild rice samples regarding γ-oryzanol contents, there were no significant differences observed in the profile (Fig. 1c; Table 1). The total amount of γ-oryzanol in MNL was 33 and 50 % higher than the amounts found in the brown rice, MGR and LGR, respectively. The amount of γ-oryzanol in MC, MNGLR, AA, S, and NOW was at least 20 % higher than the control (Table 1). On the other hand, no significant differences were observed in the total amounts of γ-oryzanol between MC, MNGLR, AA, S, NOW and the control MGR.

The results from the present study showed a significant difference in the γ-oryzanol profile between North American wild rice (*Zizania palustris*) and the regular brown rice samples (*Oryza sativa L*.). Compared to the regular brown rice, two additional isomers of cycloartenol ferulate were found in the oryzanol profile of the wild rice, representing one extra isomer over the highest number reported in the literature [12]. Whereas the amount of stigmasterol *trans*-ferulate was up to 8 times higher in wild rice compared to regular brown rice, the corresponding saturated steryl ester, stigmastanol *trans*-ferulate, amount was 10 times higher in the brown rice (Table 1). Furthermore, cycloartanol ferulate was not detected in any of the wild rice samples, whereas 14 and 54 μg/g of this compound was found in the control LGR and MGR samples, respectively. In agreement with previous studies [12, 20–22], 24-methylenecycloartanol ferulate was the most abundant steryl ferulate in the regular brown rice, LGR and MGR with the amounts up to five times higher than in the wild rice samples (Table 1). On the contrary, cycloartenol ferulate was the most abundant component in the wild rice samples, representing up to 48 % of the total amounts of γ-oryzanols (Table 1).

**Fig. 2:**
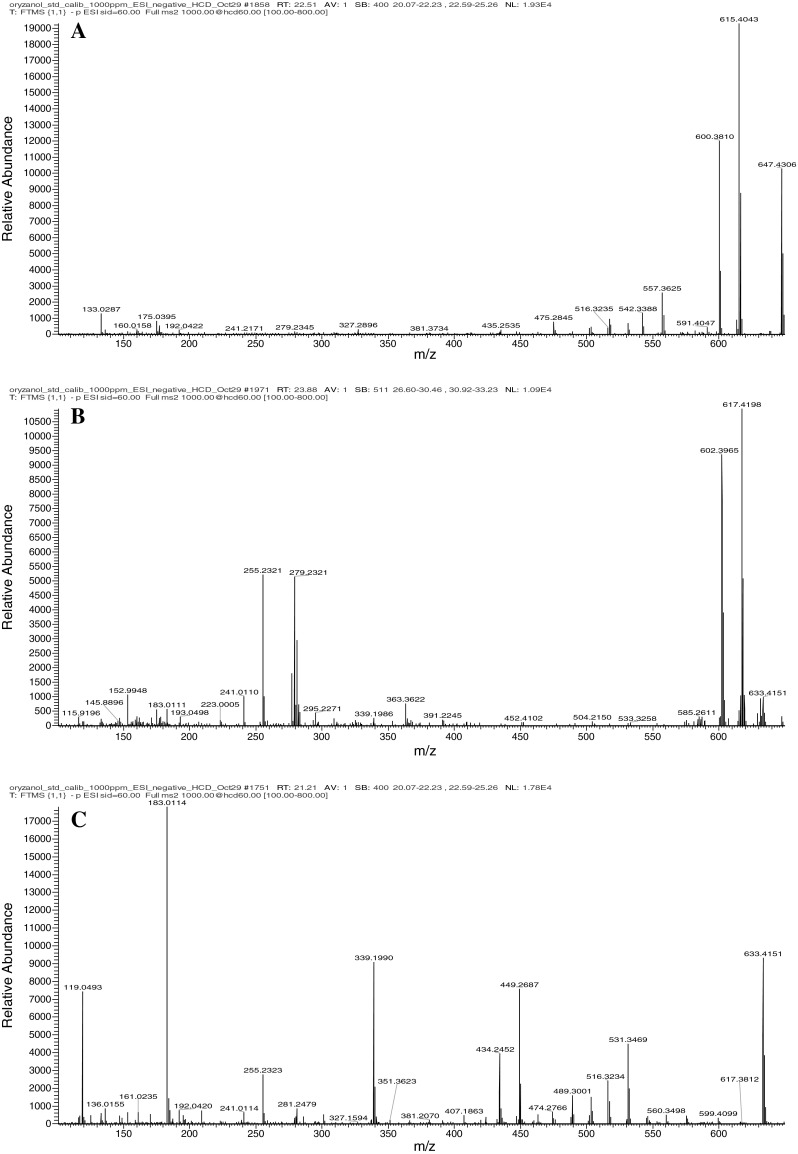
MS spectra of hydroxylated steryl ferulates identified in this study: Hydroxydehydrocycloartenol ferulate (**a**); Hydroxycycloartenol ferulate (**b**); Hydroxy-24-methylenecycloartenol ferulate (**c**)

All the seven hydroxylated steryl ferulates previously reported by Fang et al. [12] were identified in both the wild and control samples (Fig. 2). Furthermore, campesterol and cycloartenol caffeates were reported by Fang et al. [12], and in the present study, components tentatively identified as campesterol and cycloartenol caffeates (**8** and **9,** Table 1) were found in all the wild rice samples where they accounted for up to 13 % of the total γ-oryzanols amounts. Two additional peaks tentatively identified as campesterol and cycloartenol cinnamates (**21** and **22**, Table 1) were also observed in all the wild rice samples. None of these compounds were observed in the control brown rice samples, indicating a broader diversity of γ-oryzanols in the North American wild rice.

**Fig. 3:**
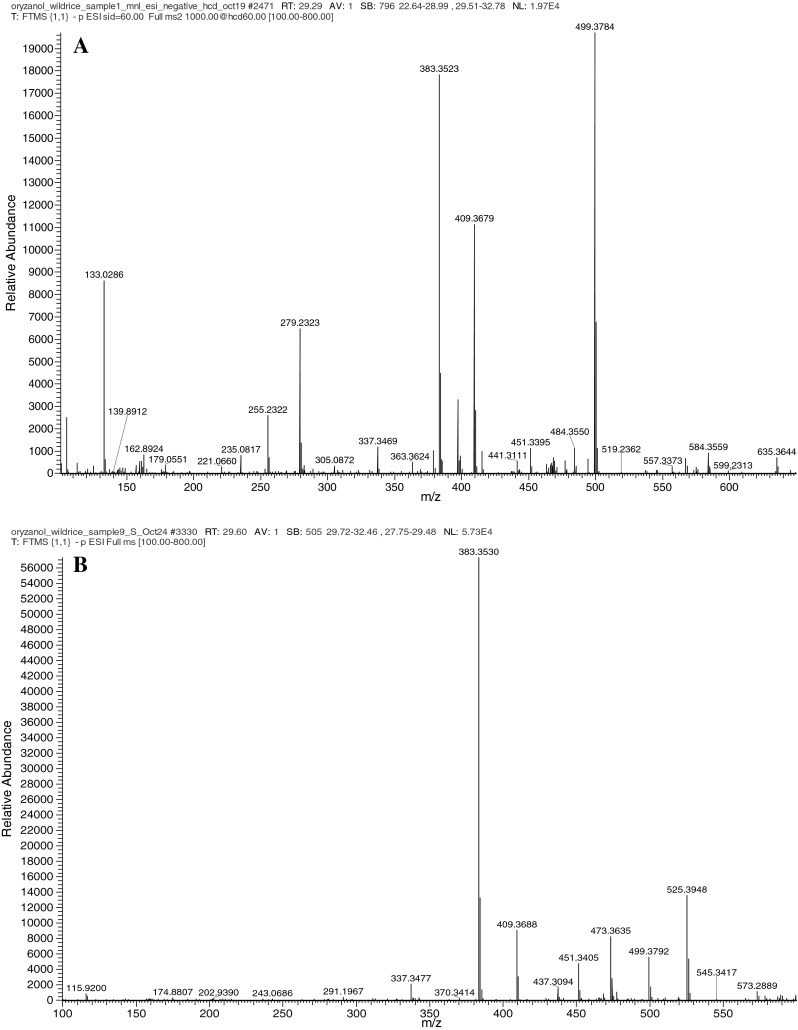
MS spectra of components tentatively identified as cycloartenol caffeate (**a**) and campesterol caffeate (**b**)

The tentative identification of peaks **8** and **9** was based on the following premises: Two of the most abundant ions in the ESI–MS spectra of compounds **8** and **9** were at *m*/*z* 409 and 383, respectively, which is essentially identical to those of the major components of cycloartenol and campesterol ferulates, respectively (Fig. 3). This data indicating that compounds **8** and **9** are respective phenolic esters of cycloartenol and campesterol. The retention time of compound **8** and **9** on the reversed phase column suggested that they are more polar than cholesterol ferulate and other major sterol ferulates in rice but less polar than the hydroxylated ferulates previously reported [12]. When connected with the fragment ion at *m*/*z* 179 suggested that compounds **8** and **9** are most likely caffeic acid esters of sterols. The sterol caffeates reported by Fang et al. also eluted between cholesterol ferulate and the more polar hydroxylated ferulates on the C18 column [12].

**Fig. 4:**
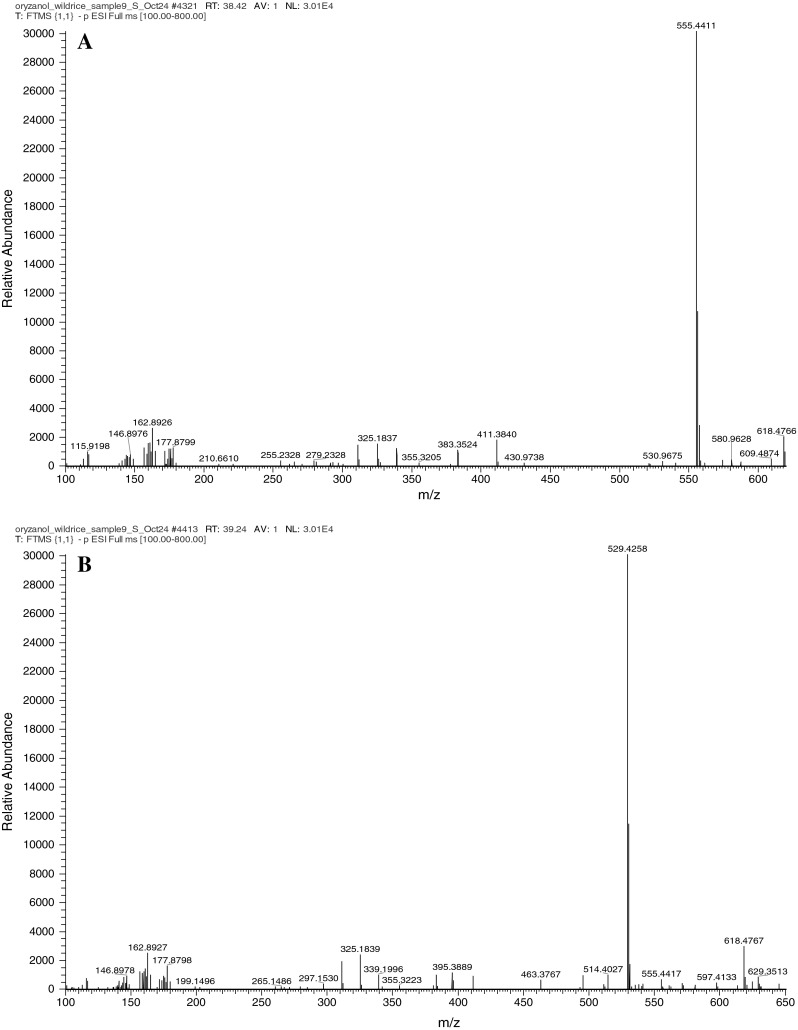
MS spectra of components tentatively identified as cycloartenol cinnamate (**a**) and campesterol cinnamate (**b**)

Compounds **21** and **22** with characteristic ions at *m*/*z* 555 and 529, respectively (Fig. 4a, b), are also unique to the wild rice. The presence of ions at *m*/*z* 410 and 383 in the ESI–MS positive mode spectrum suggested that they are likely cycloartenol and campesterol steryl esters, respectively. The mass difference of 46 amu between these compounds and the corresponding ferulates suggested the absence of methoxy and hydroxyl groups in the phenolic acid moiety and led to the tentative identification of these compounds as the cinnamate derivatives of the respective sterols.

Although there is a possibility of co-eluting glycerol esters such as mono and diacylglycerides with γ-oryzanol compounds [23], however, the fragmentation patterns of compounds **8**, **9**, **21** and **22**, and their absence in control brown rice, which are known to contain more than twice the amount of lipid than wild rice [17], suggested that these compounds are more likely γ-oryzanol compounds. Further spectrometric assessment is required to complete structural verification of these components.

## Conclusion

This study contains the first report on the γ-oryzanol profiles in the North American wild rice (*Zizania palustris*). The results from the present study showed significant differences in both composition and content between wild rice and the regular brown rice (*Oryza sativa*) L.. With four different isomers identified, cycloartenol ferulate was the most abundant sterol ferulate in the wild rice samples, whereas 24-methylenecycloartenol ferulate was the most abundant in brown rice. It appears that there are more saturated ferulates in regular brown rice compared to wild rice. Four additional γ-oryzanol compounds were tentatively identified as caffeates and cinnamates of cycloartenol and campesterol in all the wild rice, however, further study is required to confirm their chemical structure.
